# Contralateral pneumothorax with postpneumonectomy syndrome: report of 18-year-old case

**DOI:** 10.1093/icvts/ivae065

**Published:** 2024-04-12

**Authors:** Takaya Sato, Kentaro Minegishi, Shunsuke Endo, Hiroyoshi Tsubochi

**Affiliations:** Department of General Thoracic Surgery, Jichi Ika University Saitama Medical Center, Saitama, Japan; Department of General Thoracic Surgery, Jichi Ika University Saitama Medical Center, Saitama, Japan; Department of General Thoracic Surgery, Jichi Ika University Saitama Medical Center, Saitama, Japan; Department of General Thoracic Surgery, Jichi Ika University Saitama Medical Center, Saitama, Japan

**Keywords:** Postpneumonectomy syndrome, Pneumothorax, Bullectomy, Thoracoscopic surgery, Selective lobar ventilation

## Abstract

We herein report a case of an 18-year-old male with left postpneumonectomy syndrome who underwent a bullectomy for right pneumothorax. The patient underwent a left pneumonectomy at the age of 1 year. At the age of 18 years, he developed right pneumothorax, and radiological findings revealed apical bullae in the right pleural cavity extending into the left atrophic thoracic cavity beyond the upper mediastinum. The right thoracoscopic bullectomy was successful. Modifications of selective lobar ventilation during surgery and thoracoscope position were described.

## INTRODUCTION

Overextension of a remaining lung with mediastinal shift after pneumonectomy, what is called, postpneumonectomy syndrome, can lead to severe respiratory symptoms such as persistent dyspnoea, haemoptysis and cyanosis. Incidental contralateral pneumothorax is also life-threatening condition and requires urgent intervention. Less invasive bullectomy for the patient with postpneumonectomy syndrome is challenging.

We report a case of an 18-year-old patient who underwent a thoracoscopic bullectomy for contralateral pneumothorax with the postpneumonectomy syndrome.

## ETHICAL STATEMENT

We affirm this research adheres to the ethical standards and guidelines set by Jichi Ika University Saitama Medical Center and has received approval from IRB on 12 October 2023. The IRB approval number is S23-073.

## CASE

The patient was an 18-year-old male who underwent a left lower lobectomy for congenital pulmonary sequestration at the age of 1 year. The left completion pneumonectomy was performed after postoperative torsion of the left upper lobe. He had been followed for postpneumonectomy syndrome, but only observation due to lack of symptoms. His other medical history included scoliosis and gender identity disorder.

**Figure 1:**
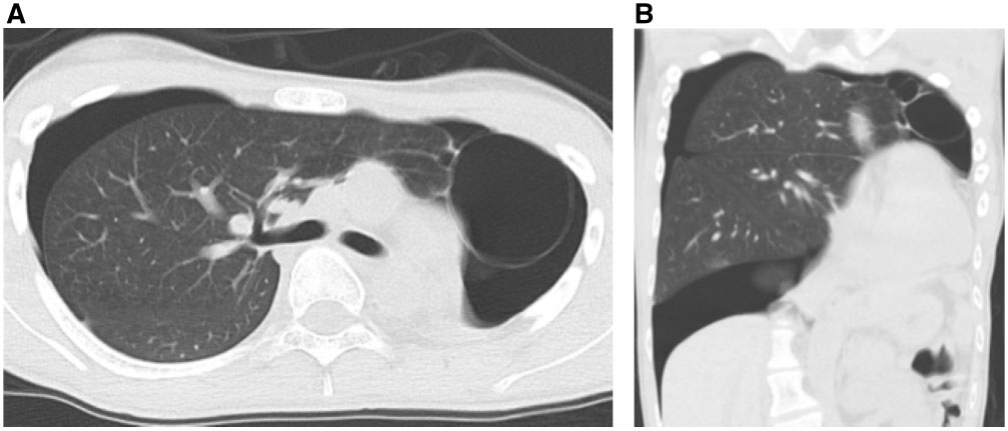
Chest computed tomography showed right pneumothorax after left pneumonectomy. Note that apical bullae located in the left atrophic chest cavity due to the overextension of the right pleural cavity beyond the upper mediastinum.

**Figure 2:**
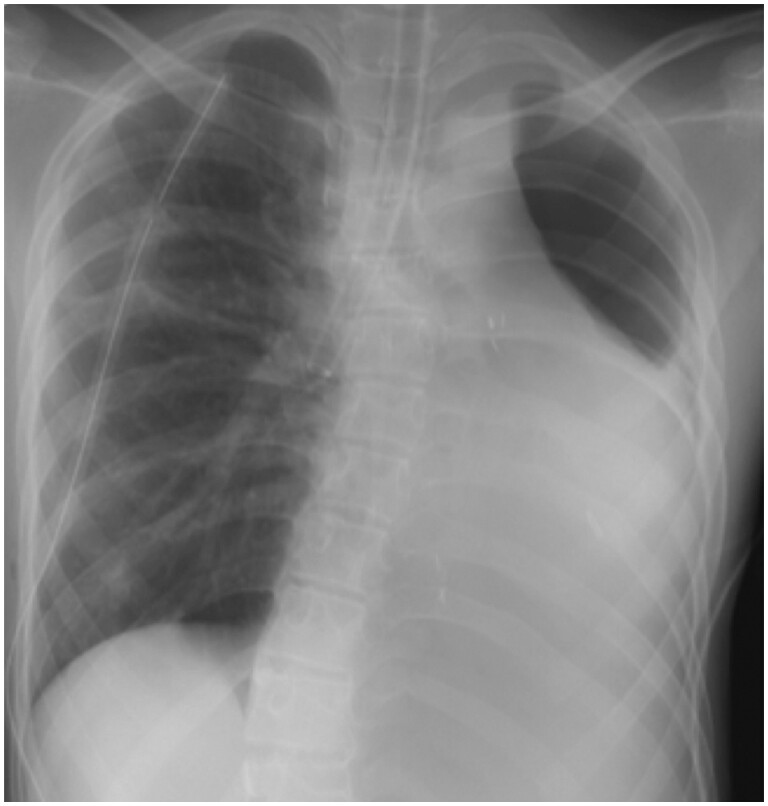
Chest radiography during surgery showed the tip of the left-sided double-lumen endobronchial tube in the right intermediate bronchus for selective lobar ventilation.

He presented to our hospital with a complaint of sudden severe dyspnoea. Computed tomography revealed a right pneumothorax and apical bullae protruding towards the apex of the left atrophic chest cavity (Fig. 1). The right pneumothorax with the left postpneumonectomy syndrome was diagnosed and he had a chest tube inserted. Due to the persistent air leakage from the chest drain, surgical intervention was performed on the 3rd day of hospitalization. The patient underwent a right thoracoscopic bullectomy under general anaesthesia in the supine position. For selective lobar ventilation of the right middle/lower lobes (Fig. 2), the tip of the left-sided double-lumen endobronchial tube was placed in the right intermediate bronchus under bronchoscopic guidance. During the right thoracoscopic bullectomy, the scope was inserted via the left chest cavity to provide a surgical view of the protruded apical bullae towards the left thoracic cavity beyond the upper mediastinum. A thoracoscopic bullectomy was successful. Operation time was 80 min. The postoperative course was uneventful, with drain removal on the 2nd postoperative day and discharge on the 4th postoperative day. No recurrence or complications were observed at 6 months follow-up.

## DISCUSSION

Postpneumonectomy syndrome is a potentially serious complication that can occur after a pneumonectomy surgery. It is a rare syndrome, with an estimated prevalence of ∼1 in 650 pneumonectomies. Postpneumonectomy syndrome is generally more prevalent and severe after right pneumonectomy, between the spine and the aorta posteriorly, as well as the pulmonary artery anteriorly. On the other hand, the course of postpneumonectomy syndrome following left pneumonectomy is generally mild, often allowing for conservative treatment, as in this case. It is more common in patients who undergo surgery in childhood because of increased elasticity and compliance of the remaining lung and bronchial tree [1]. Treatment options include repositioning the mediastinum, filling the postpneumonectomy space with a non-absorbable material and endobronchial stenting [2].

Contralateral pneumothorax after pneumonectomy is also a rare and life-threatening condition. The overall mortality rate is reported to be ∼50% [3]. Given the lethality of this condition, we must consider treatment options to prevent recurrence, such as surgery and pleurodesis [4]. Pleurodesis is a common and effective treatment for pneumothorax. However, in this case, the effect of pleurodesis may not be promising because the lung bulla protruded into the contralateral chest cavity.

Thoracoscopic bullectomy for the patient with postpneumonectomy syndrome is challenging in terms of oxygenation during surgery. Selective lobar ventilation technique is not suitable for patients with compromised respiratory function or emphysematous lungs due to poor visualization in video-assisted thoracoscopic surgery. On the other hand, extracorporeal membrane oxygenation is an effective way to maintain oxygenation in patients with compromised respiratory function, but there is a potential issue of bleeding complications due to the need for anticoagulant [5]. In this case, because the patient had preserved respiratory function and no emphysema, we chose a selective lobar ventilation technique, which is less invasive and has a lower risk of bleeding.

Lobar isolation using a bronchial blocker is well known. However, in this post-left pneumonectomy case, a left-sided double-lumen endobronchial tube was inserted and the tip of the tube was placed in the right intermediate bronchus to collapse only the right upper lobe and prevent hypoxemia. This method is simpler and can collapse the lobes more effectively than using a blocker, but if the left-sided double-lumen tube is not rotated 180° when inserted to the right intermediate bronchus, the tip of the tube may hit the bronchial wall, resulting in inadequate ventilation.

Moreover, surgery was performed in the supine position to facilitate rapid introduction of extracorporeal membrane oxygenation in case of failure of selective lobar ventilation. Additionally, a port was created in the contralateral chest cavity to insert a thoracoscope for visualization of bullae protruding to the contralateral side.

The combination of these maneuvres was a unique approach and allowed for minimally invasive surgery in this challenging case.

## CONCLUSIONS

Contralateral pneumothorax after pneumonectomy with postpneumonectomy syndrome is a life-threatening and surgically challenging condition. Modifications of ventilation and thoracoscope insertion could make a safe and easy video-assisted thoracoscopic surgery.

## Data Availability

The data that support the findings of this study are available from the corresponding author, Takaya Sato, upon reasonable request.
